# Strain-specific transmission in an outbreak of ESBL-producing Enterobacteriaceae in the hemato-oncology care unit: a cohort study

**DOI:** 10.1186/s12879-016-2144-4

**Published:** 2017-01-05

**Authors:** Makiko Uemura, Osamu Imataki, Shumpei Uchida, Haruyuki Nakayama-Imaohji, Yukiko Ohue, Harumi Matsuka, Hatsune Mori, Hiroaki Dobashi, Tomomi Kuwahara, Norimitsu Kadowaki

**Affiliations:** 1 Division of Hematology, Rheumatology and Respiratory Medicine, Department of Internal Medicine, Faculty of Medicine, Kagawa University, 1750-1 Ikenobe, Miki-cho, Kita-gun, Kagawa 761-0793 Japan; 2 Division of Molecular Microbiology, Kagawa University, Kagawa, Japan; 3 Nursing Division, Kagawa University Hospital, Kagawa, Japan

**Keywords:** Extended-spectrum β-lactamase (ESBL), *Klebsiella pneumoniae*, *Escherichia coli*, Non-clonal outbreak, Multidrug resistance

## Abstract

**Background:**

Extended-spectrum β-lactamase (ESBL)-producing bacteria are resistant to several types of antibiotics excluding carbapenems. A transmissibility of ESBL-producing Enterobacteriaceae would be depending on each bacterial property, however, that has not been elucidated in clinical setting. In this study, we attempted to identify the source of an outbreak of ESBL-producing bacteria in a medical oncology and immunology care unit.

**Methods:**

An ESBL-producing Enterobacteriaceae (ESBL-E) outbreak observed between July 2012 and August 2012 in Kagawa University Hospital was surveyed using various molecular microbiology techniques. We used Pulsed-field gel electrophoresis (PFGE), PCR-based ESBL gene typing, and direct sequence of ESBL gene as molecular microbiology typing method to distinguish each strain.

**Results:**

The typical prevalence of ESBL-E isolation in the unit was 7.0 per month (1.7 per week). The prevalence of ESBL-E isolation during the target research period was 20.0 per month (5.0 per week). In total, 19 isolates (11 *K. pneumoniae* and 8 *E. coli*) were obtained from clinical samples, including four control strains (two each of both bacteria), that were physically different from those obtained from other inpatient units in our hospital. Pulsed-field gel electrophoresis (PFGE) for *K. pneumoniae* (digested by *Xba*I) produced similar patterns excluding one control strain. PCR classification of the ESBL gene for *K. pneumoniae* revealed that all strains other than the control strain carried SHV and CTX-M-9. This result was reconfirmed by direct DNA sequencing. Although the outbreak of *K. pneumoniae* was considered to be “clonal,” PFGE and PCR classification of the ESBL genes for *E. coli* uncovered at least six different “non-clonal” strains possessing individual ESBL gene patterns. According to the result of an antibiogram, the pattern of antimicrobial susceptibility was more variable for *K. pneumoniae* than for *E. coli*.

**Conclusions:**

Typing by PFGE and ESBL gene PCR analysis is practical for discriminating various organisms. In our cohort, two outbreaks were concomitantly spread with different transmission strategies, namely clonal and non-clonal, in the same unit. This might represent clinical evidence that transmissibility differs according to the type of strain. We speculated that patient-to-patient transmission of ESBL-E occurred according to the properties of each individual strain.

**Electronic supplementary material:**

The online version of this article (doi:10.1186/s12879-016-2144-4) contains supplementary material, which is available to authorized users.

## Background

Extended-spectrum β-lactamase (ESBL)-producing bacteria are resistant to most beta-lactam antibiotics, including penicillins, cephalosporins, and the monobactam. Consequently, infections caused by ESBL-producing bacteria are difficult to treat. In addition, there are no evidence-based guidelines specifying the infection control for ESBL-producing bacterial outbreak. Therefore, ESBL-producing bacteria easily disseminate and cause nosocomial infection. Infection control of ESBL-producing bacteria is important to prevent outbreaks [1].

An outbreak is defined as the increased incidence of an infectious disease in a specific place during a given period that exceeds the baseline rate for that place and period [2]. According to past reports, outbreaks of ESBL-producing bacteria develop from a single source of infection related to a unique original source, termed “clonal” outbreaks [3]. However, “clonal” outbreak is not the dominant dissemination pattern in limited areas such as care units in healthcare institutes. Global propagation of ESBL genes has been reported among carriers of ESBL-producing bacteria in the community, and it is becoming a general form of bacterial spread [4–6]. In other words, outbreaks of ESBL-producing bacteria can be caused by multiple sources, termed “non-clonal” outbreaks. Opposed to the clonal spread of ESBL-producing bacteria, non-clonal outbreaks are becoming more common [7–9]. In addition to the clonality of outbreak strains, the molecular mechanism of ESBL gene acquisition may contribute to the dynamics of transmission in the clinical setting [8, 10]. However, differences in the spread of ESBL-producing bacteria have not been fully unveiled.

In our institution, we experienced an outbreak of ESBL-producing bacteria in our medical oncology and immunology care unit. We attempted to identify the original strains of the first index case of infection to clarify the source of infection outbreaks and control nosocomial spread; we performed molecular microbiological analysis of ESBL-E bacteria in our retrospective cohort. The outbreak comprised a mixture of two types of ESBL-producing Enterobacteriaceae (ESBL-E), *Klebsiella pneumoniae* and *Escherichia coli*. A main interest of this research is the mechanism of spread of ESBL-E. Our hypothesis is ESBL-producing bacteria has a different transmissibility based on different bacterial properties.

## Methods

### Patients and method

Between July and August 2012, in the medical oncology and immunology care unit of our institute (41 beds), 19 strains were obtained from clinical samples during ESBL-E outbreaks. Strains obtained in the unit included the samples of all patients who participated in surveillance during the observed outbreaks. Control strains were prepared from clinically different isolates from heterotopy/heterochrony sampling (labeled as © in Figures). We used the first isolate from each clinical sample of all individuals for the analysis. Thus, two or more isolates could be obtained from each individual, such as one strain from stool and the other from blood. Our target species were ESBL-E, namely *K. pneumoniae* and *E. coli* [8]. Although we observed the outbreak in 2012, the institutional review board approved our research in 2015.

In this study, we defined two outbreak types according to the source of infection as follows: 1) outbreak from a single source (clonal outbreak) and 2) outbreak from multiple sources (non-clonal outbreak). An outbreak is defined as an increase in the rate of ESBL-E cases or a clustering of new cases in a specific place during a given period. In this report, we defined an unusual increase in ESBL cases as a repeated isolation of ESBL from the medical oncology and immunology care unit, with an incidence ±2 SD over the baseline.

### Molecular microbiology methods

#### Pulsed-field gel electrophoresis (PFGE) typing

Strains isolated from clinical samples were pulsed-electrophoresed on a degraded field agarose gel. The Tenover criteria were used for the separation and identification of each band [2]. Only strains with indistinguishable band profiles were considered to represent the same clone. ESBL-producing *K. pneumoniae* and *E. coli* isolates were aerobically cultured in brain heart infusion broth (Eiken Chemical Co. Ltd.) for 16 h at 37 °C. PFGE plugs were prepared using a Gene Path Kit (Bio-Rad) according to the manufacturer’s instructions. The plugs were digested overnight with 30 U of *Xba* I (New England Biolabs) at 37 °C. The digested DNA bands were separated on 1.0% agarose gels by PFGE using the CHEFF DR II system (Bio-Rad). PFGE was performed under the following conditions: electric field strength, 6 V/cm; pulse time, 4–8 s for 9 h followed by 8–50 s for 13 h; and buffer temperature, 14 °C. After electrophoresis, the gels were stained with ethidium bromide (0.5 μg/ml).

#### Antimicrobial susceptibility testing

The antibiotic susceptibility of strains isolated from clinical samples was assessed by microdilution methods using an IA20MIC mkII system (Koden Industry Co., Japan). The following antibiotics were used: piperacillin/tazobactam (PIPC/TAZ), cefotiam (CTM), ceftazidime (CAZ), cefoperazone/sulbactam (CPZ/SBT), cefpirome (CPR), azactam (AZT), minocycline (MINO), imipenem/cilastatin (IPM/CS), meropenem (MEPM), ciprofloxacin (CPFX), levofloxacin (LVFX), and amikacin (AMK). Breakpoints were adapted according to Clinical and Laboratory Standards Institute (CLSI) criteria. Susceptibility was determined by disc diffusion, following the CLSI recommendations for Enterobacteriaceae (CLSI 2010, Performance Standards for Antimicrobial Susceptibility Testing; Twentieth Informational Supplement, M100-S20, Jan. 2010). Drug resistance among the ESBL-producing isolates was assessed by disk dilution methods. Evidence of ESBL production was defined as synergy between co-amoxiclav and at least one of the following antibiotics: cefotaxime (CTX), CAZ, or CPFX. The minimum inhibitory concentrations of CTX and CAZ, with and without clavulanic acid (CVA), were determined subsequently [11].

#### PCR-based ESBL gene typing

Molecular ESBL typing was performed as described previously [12]. In detail, ESBL-producing bacteria were screened by PCR/DNA for the detection of *bla*_TEM_, *bla*_SHV_, and *bla*_CTX-M_. Subsequently, ESBL genes with specific CTX-M subtypes (groups 1, 2, 8 and 9) were characterized using multiplex PCR amplification of the DNA extracted from each strain as previously described [13].

### Data collection and ethical issues

We retrospectively obtained patient data from their medical records. This cohort study was conducted under the approval of the institutional review board (IRB) of our institute (IRB approval No. 27-197).

We numbered the strains as an identifier number which does not compromise patient anonymity.

## Results

**Fig. 1:**
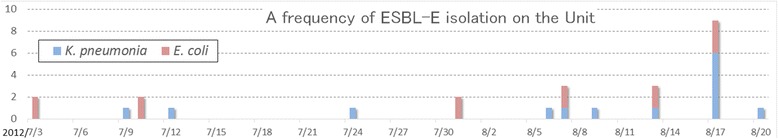
Frequency of extended-spectrum β-lactamase-producing Enterobacteriaceae (ESBL-E) isolation in our unit. The incidence of ESBL-E isolation in the microbiological laboratory in our institute was determined. *Klebsiella pneumoniae* is indicated by the gray bar, and *Escherichia coli* is denoted by the shadow bar. Only the first microbe isolated from each sample was counted, but the isolation of different specimens in different samples from the same individual was permitted

**Fig. 2:**
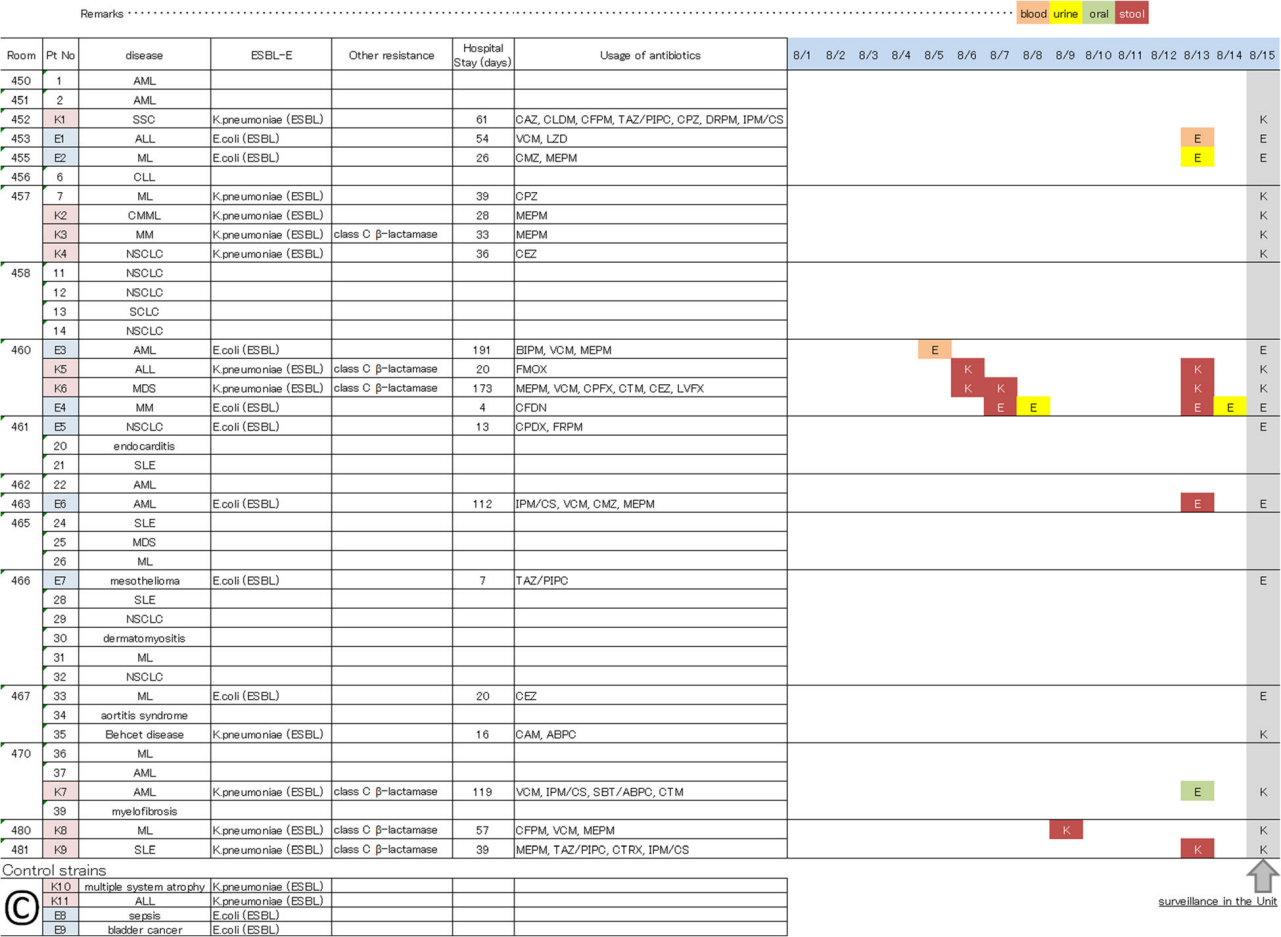
A description of the patients and the times at which extended-spectrum β-lactamase-producing Enterobacteriaceae (ESBL-E) were isolated in our unit. Patients’ characteristics (age, gender, and diseases) are shown. A unique strain number was provided according to the room number. “K” denotes *K. pneumoniae* isolates, and “E” indicates *E. coli* isolates. The types of clinical specimens (blood, urine, oral, and stool) are indicated by red, yellow, green, and brown boxes, respectively. The four control strains, comprising clinically distinct isolates, are shown at the bottom of the list. The rightmost row (row in dark) presents the results of surveillance performed at the end of the outbreak

The typical prevalence of ESBL-E isolation in the unit was 7.0 per month (1.7 per week). The prevalence of ESBL-E considered positive instances at all the infection sites. A different isolate from the same sampling site in the same individual was not counted double, but a different isolate from the different sampling site in the same individual was counted as a different event. The prevalence of ESBL-E isolation during the target research period (July to August 2012) was 20.0 per month (5.0 per week; Fig. 1). We recognized this is an outbreak of ESBL-E in the given period. The time course of ESBL-E isolation in the room is depicted in Fig. 2. Total 19 patients were affected by the outbreak and 15 isolates were used for our analysis. The patients involved in this study were 16 females and 3 males. The median age was 65 years (range 25–73 years). The patients’ diseases and isolation samples were shown in Fig. 2. The median duration from admission to the day of ESBL-E detection was 36 days (range 4–191 days). A cluster outbreak was identified in Room 480, but it was merely a partial phenomenon. The regional outbreak in Room 480 was assumed to be due to transmission. The patients in Room 480 shared a bathroom.

**Fig. 3:**
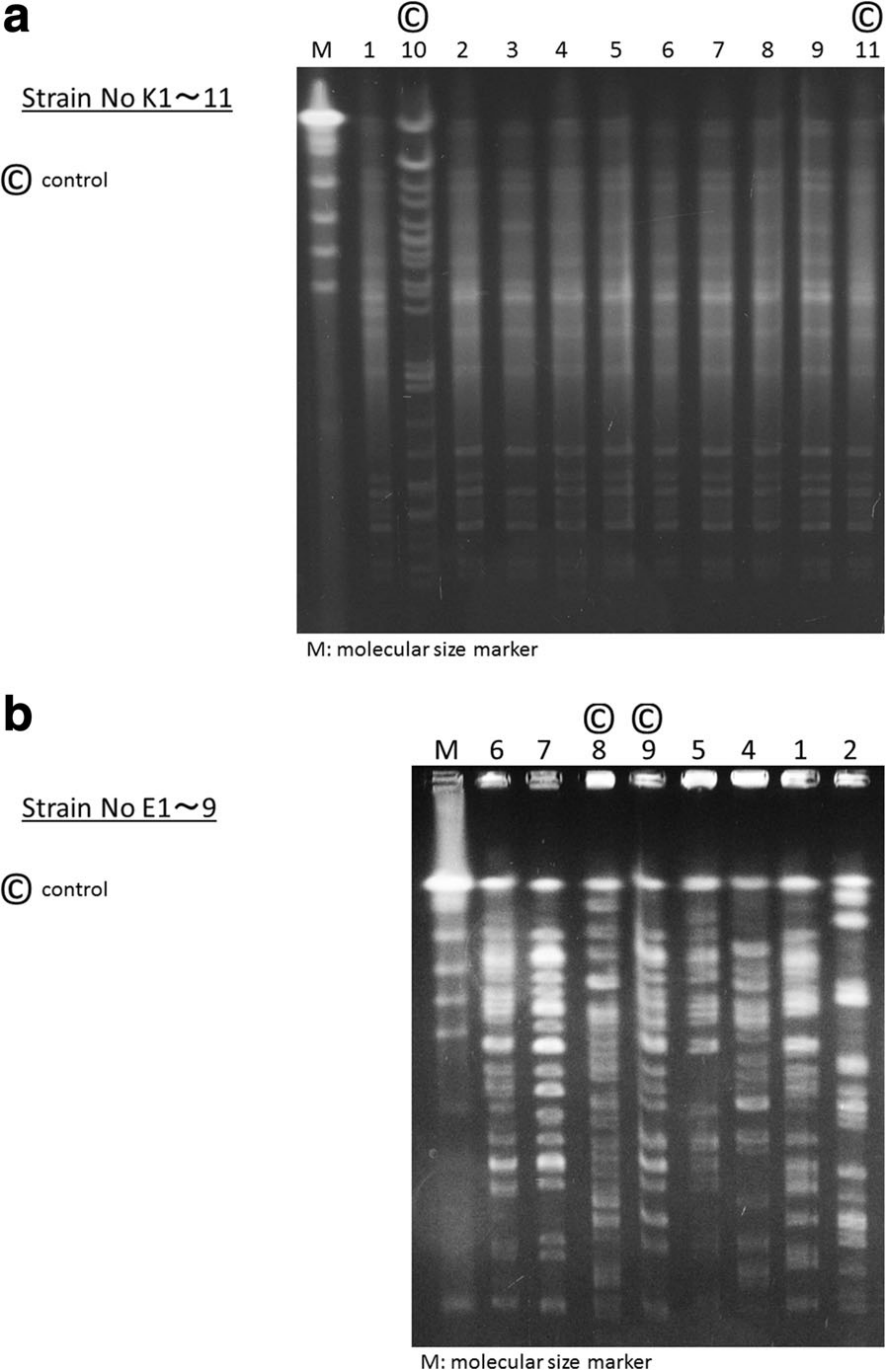
Pulsed-field gel electrophoresis patterns of the genomic DNA of extended-spectrum β-lactamas-producing Enterobacteriaceae The left lane presents the molecular markers. **a** *Klebsiella pneumoniae*: The unique strain numbers K10 and K11 denote control strains (labeled as ©). **b** *Escherichia coli*: The unique strain numbers E8 and E9 denote control strains (labeled as ©). The strain E3 was missing. Control strains were prepared from clinically different isolates from heterotopy/heterochrony sampling

PFGE patterns could not discriminate a series of nine *K. pneumoniae* strains from clinical samples (with two control strains, K10 and K11; Fig. 3a). However, regarding *E. coli*, all six strains were discriminated as different strains (with 2 control strains, E8 and E9, Fig. 3b). Next we examined the susceptibility of sample strains. In addition, the results of ESBL gene typing were compatible with those of PFGE typing. All ESBL genes extracted from *K. Pneumoniae* were categorized in CTX-M-9 excluding one control strain (K10) which had carried SMV (Table 1). On another, ESBL genes derived from *E. coli* strains were varied in each strain with TEM, SMV, CTX-M-1, CTX-M-2, or CTX-M-9 (Table 1). The result of an antibiogram were supplied in the supplemental data (Additional file 1: Table S1). The pattern of antimicrobial susceptibility was more variable for *K. pneumoniae* than for *E. coli*.

**Table 1:** Typing of the extended-spectrum β-lactamase gene

a								
Strain ID	Patient ID	Species	TEM	SHV	CTX-M-1	CTX-M-2	CTX-M-8	CTX-M-9
K1	17868	Klebsiella pneumoniae (ESBL)						○
K10	2439551	Klebsiella pneumoniae (ESBL)		○				○
K2	2607183	Klebsiella pneumoniae (ESBL)						○
K3	1001542	Klebsiella pneumoniae (ESBL)						○
K5	3082844	Klebsiella pneumoniae (ESBL)						○
K6	3083200	Klebsiella pneumoniae (ESBL)						○
K7	2014171	Klebsiella pneumoniae (ESBL)						○
K7	3052387	Klebsiella pneumoniae (ESBL)						○
K8	3073729	Klebsiella pneumoniae (ESBL)						○
K9	1824592	Klebsiella pneumoniae (ESBL)						○
K11	932245	Klebsiella pneumoniae (ESBL)						○
b								
Strain ID	Patient ID	Species	TEM	SHV	CTM-1	CTM-2	CTM-8	CTM-9
E1	2021190	Escherichia coli (ESBL)						○
E2	2535581	Escherichia coli (ESBL)						○
E3	1731148	Escherichia coli (ESBL)	○			○		
E4	2536875	Escherichia coli (ESBL)	○					○
E5	2304303	Escherichia coli (ESBL)						○
E6	3065667	Escherichia coli (ESBL)						○
E7	2356293	Escherichia coli (ESBL)						○
E8	2362963	Escherichia coli (ESBL)	○			○		
E9	2568787	Escherichia coli (ESBL)	○		○			

Multiplex PCR results for TEM, SMV, CTX-M-1, CTX-M-2, CTX-M-8, and CTX-M-9 are presented. a) *Klebsiella pneumoniae*: All strains carried CTX-M-9, excluding one control strain (K10) that carried SMV. b) *Escherichia coli*: Excluding E1, E2, E5, E6, and E7, all strains carried different types of ESBL genes. The strain identifier numbers presented in the table were not linked to patient’s privacy and cannot compromise patient anonymity

In totality, the results of PFGE, antimicrobial susceptibility testing, and ESBL gene typing were not consistent for *K. pneumoniae* and *E. coli*. All isolates from *K. pneumoniae* belonged to sequence type (ST) 1308, where control strain (K2) harbored ST1728. This result was consistent with the ESBL gene subtypes (CTX-M-9 identified in *K. pneumoniae* isolates in our cohort). The outbreak of ST1308 was determined genetically. The antibiogram pattern varied for *K. pneumoniae*. The outbreak of ESBL-producing *K. pneumoniae* could be judged to have the same source of infection in some patients, although not all cases in this episode involved infection with the same strain. For instance, in Room 480, cluster clonal transmission could have easily occurred. Of note, case K7 harbored *K. pneumoniae* and *E. coli* in the oral cavity and stool, respectively. On the other hand, the isolates from *E. coli* were genetically different by PFGE and ESBL gene typing. The outbreak of ESBL-producing *E. coli* strains did not disseminate from the same source of infection as *K. pneumoniae*. Thus, we concluded that a mixed clonal and non-clonal outbreak had occurred in each strain, respectively.

## Discussion

The distinction of strain clonality by antimicrobial susceptibility testing is difficult. Resistance to multiple classes of antibiotics is described as multidrug resistance. Although ESBL exerts a distinctive mechanism for multidrug resistance, subclasses of multidrug resistance have not been elucidated. Thus, our original categorization of drug resistance in this study is not essential, and we considered the antibiogram result to be supplemental data. Conversely, PFGE and ESBL gene type was conducted for ESBL-E strain identification, and we obtained solid results. These analyses of our cohort identified molecular similarity in the PFGE patterns of ESBL-producing *K. pneumoniae*. Although one of the control strains and clinically different strains were not distinguishable, we realized that *K. pneumoniae* strains were of the same origin in the cohort. Identification of the source of infection only based on antimicrobial susceptibility testing in the clinical laboratory would not be practical. Given the dissemination of ESBL-producing bacteria and ESBL gene spread in the community, tracing the source of infection in ESBL-E outbreaks is not beneficial in practice. In any case involving ESBL-producing bacteria, standard precautions should be taken, but tracing the original index case would not contribute to clinical decisions.

An obvious source of environmental contamination can be easily detected. In our cohort, the clonal outbreak was associated with fecal spread in the bathroom. To estimate the clonality of an outbreak, the location of the outbreak is important. Detecting the ESBL source, i.e., “looking for the culprit,” may support in infection-control measures, but it is impossible to achieve complete patient isolation immediately [10]. Patient-to-patient transmission of ESBL-E via the hands of healthcare workers in our unit appeared to be common. Therefore, concomitant outbreaks can occur in a nosocomial setting in which most patients carry or display contamination with ESBL-E. Indeed, our surveillance at the end of the outbreak revealed that 46.3% (19/41) of patients were colonized or infected with ESBL-E (Fig. 2). In multifocal outbreaks, efforts to detect the ESBL source will sometimes end in failure.

Numbers of reports describing clonal outbreak arising from a single source have been published [3–6]. ESBL-E commonly reside in the intestine [10, 14]. Generally, ESBL-E spread from the digestive tract via direct contact, including that with medical items [15]. A hypothesis explaining the unique infection source was not conceivable in our cohort. Yet, in some cases, commonly occurring outbreaks are due to non-clonal expansion. A community-acquired methicillin-resistant *Staphylococcus aureus* (MRSA) infection is increasing, and the same is for ESBL-E. MRSA, both in the community and hospital settings, colonizes and has low virulence [16]. And ESBL-E can also colonize, and in such cases, it does not become pathogenic in healthy individuals. Some observers in our hospital laboratories, nevertheless, emphasized on food-borne delivery as the source of outbreak merely by referencing a rare report [12]. In accordance with the decision of the infection control team (ICT), they prepared a culture of frozen food in a backward manner to the onset of the outbreak. No ESBL-E bacteria were detected and the ICT ended to conclude community-acquired ESBL-E is a main source of outbreak. Emerging spreads of ESBL-producing bacteria in the community is currently a dominant pattern of infection [4].

In this study, we characterized the different penetrance patterns of the two species of ESBL-E. Our result suggests that these two bacterial species obtained the ESBL gene according to their microbiological properties [1]. PFGE demonstrated that 10 of 11 *K. pneumoniae* isolates were similar in our analysis. *K. pneumoniae* isolates in our cohort possessed the CTM-X-9 type of the ESBL gene. *bla*_CTM-X_ has been known to be easily mobilized into plasmids in environmental bacteria [17]. At present, the emergence of community-acquired ESBL-E infections is globally associated with the CTM-X type of ESBL [4], especially with CTM-X-9 in East Asian countries including Japan [18]. CTX-M group ESBL genes are assumed to have more facilitated spread than TEM and SHV group ESBL genes under broad-spectrum antimicrobial selection pressure. Indeed, CTX-M enzymes have rapidly supplanted TEM and SHV group ESBL genes [4]. Some microbiological advantages, which are not yet understood, might possibly contribute to the acquisition of ESBL genes in *K. pneumoniae* rather than in *E. coli*. On the contrary, in *E. coli*, ESBL genes are acquired through vector-transporting bacteriophages. Among our cases, *E. coli* possessed various ESBL genes categorized as TEM, SHV, and CTX-M types.

The digestive tract is the main reservoir for the colonizers of community-acquired ESBL-E, especially *E. coli* [15]. The probable mechanism of this variety of ESBL genes is intraluminal transmission of resistance genes in the individual gut. In our cohort, surveillance during the outbreak identified *E. coli* among the colonized patients, providing evidence of fecal carriage. Our results proved that the *E. coli* strains had genetically variable ESBL genes. Thereby, the *E. coli* outbreak was presumed to be derived from originally community-acquired strains. In general, ESBL genes are mainly transferred in a plasmid-mediated manner. ESBL genes have been reported most frequently in *K. pneumoniae* and *E. coli* [19]. Our speculation is that the ability to acquire ESBL genes differs among bacteria, and the penetrance of resistance is strain-dependent. Specifically, ESBL-E outbreaks can occur concurrently, as observed in this study. Subsequently, some non-clonal outbreaks can occur coincidentally by the non-clonal strains derived from the community colonizers. Back to the principle of infection control, upon the hypothesis that patient populations in which isolates are genetically similar most likely acquired the organism via patient-to-patient transmission, standard precaution and containment procedures should be enforced irrespective of whether the outbreak is clonal or non-clonal.

Our study has some limitations. First, this is a single-institution observation study, and the sample size is small. Hence, our observation may not represent a universal occurrence. Therefore, other similar clinical data should be accumulated. We were exclusively interested in the difference in the penetrance of the ESBL gene during an outbreak in the clinical setting. The clonal or non-clonal spread of ESBL-E is attributable to microorganisms and individual types of ESBL genes. Second, our study included isolates from both infected and colonized subjects. This protocol might confound the study conclusion. In this study, we investigated the identification of infection source including colonizing strains, in order to prevent an outbreak when the outbreak is spreading and initial action is required immediately. Third, the last limitation of the study concerns the comprehension of the control strains. We selected some independent strains isolated in other wards at different times as control samples for PFGE typing. However, one of the control strains could not be discriminated from other *K. pneumoniae* strains. Despite some environmental contamination, there was no evidence of transmission from patients known to be colonized or infected with ESBL-producing *K. pneumoniae* and *E. coli* who were discharged from the hospital or admitted to other wards.

## Conclusions

We observed that clonal and non-clonal outbreak of ESBL-E corresponding to the difference of bacteria. We were unable to identify the common source of the outbreak among the two organisms, i.e., *K. pneumoniae* and *E. coli*, in the cohort. These two organisms would possibly transmit ESBL genes according to each bacteria’s biological ability. This molecular microbiological difference did not appear in the mapping of ESBL-E spread, but it did reflect organism-specific outbreaks whether clonal or non-clonal. Our findings provide an outlook of the concurrent expansion of clonal and non-clonal outbreaks. Our cohort represents an endeavor to detect the index case of the outbreak and clarify that a subject harboring ESBL-E is no longer essential in the era of widespread ESBL-E dissemination in the community. Thus far, we confirmed the importance of substantial standard precautions in the background of the expansion of multidrug-resistant organisms in the community.

## Additional file

Additional file 1: Table S1. Antibiogram of extended-spectrum β-lactamase-producing Enterobacteriaceae. The susceptibility of each strain for the given antibiotics is presented in the table as the susceptibility titer number. The number are shown as minimum inhibitory concentration (MIC) μg/mL. Resistance is indicated by bold characters. Different clones from the same specimen in the same individual were numbered using subnumbers, e.g., XX_1 and XX_2. We classified the clones into resistance groups as follows: Group A, sensitive to all antibiotics excluding cephalosporins (e.g., CAZ); group B, resistant to PIPC and cephalosporins; group C, resistant to quinolones and cephalosporins; and group D, resistant to all antibiotics excluding carbapenems. Based on our original grouping criteria, the strains could be categorized to four resistance groups. We categorized the strains in the four groups and attempted to comprehend the clonality of each bacterium. (a) *Klebsiella pneumoniae*: K3, K4, K8, and K11 were classified into group A; K5, K7, and K9 into group B; K2 and K10 into group C; and K6 into group D. (b) *Escherichia coli*: E2 was categorized in group A, and the other strains were categorized in group D. Thus, the pattern of antimicrobial susceptibility was more variable for *K. pneumoniae* than for *E. coli*. (The control strains are remarked as ©.). The strain identifier numbers presented in the table were not linked to patient’s privacy and cannot compromise patient anonymity. (DOCX 19 kb)
