# Supplementary material for: Strain-specific transmission in an outbreak of ESBL-producing Enterobacteriaceae in the hemato-oncology care unit: a cohort study
Source: BMC Infect Dis. 2017 Jan 5;17:26. doi: 10.1186/s12879-016-2144-4 (PMC5217410; doi:10.1186/s12879-016-2144-4)
Supplement: Additional file 1: Table S1. — Antibiogram of extended-spectrum β-lactamase-producing Enterobacteriaceae. The susceptibility of each strain for the given antibiotics is presented in the table as the susceptibility titer number. The number are shown as minimum inhibitory concentration (MIC) μg/mL. Resistance is indicated by bold characters. Different clones from the same specimen in the same individual were numbered using subnumbers, e.g., XX_1 and XX_2. We classified the clones into resistance groups as follows: Group A, sensitive to all antibiotics excluding cephalosporins (e.g., CAZ); group B, resistant to PIPC and cephalosporins; group C, resistant to quinolones and cephalosporins; and group D, resistant to all antibiotics excluding carbapenems. Based on our original grouping criteria, the strains could be categorized to four resistance groups. We categorized the strains in the four groups and attempted to comprehend the clonality of each bacterium. (a) Klebsiella pneumoniae: K3, K4, K8, and K11 were classified into group A; K5, K7, and K9 into group B; K2 and K10 into group C; and K6 into group D. (b) Escherichia coli: E2 was categorized in group A, and the other strains were categorized in group D. Thus, the pattern of antimicrobial susceptibility was more variable for K. pneumoniae than for E. coli. (The control strains are remarked as ©.). The strain identifier numbers presented in the table were not linked to patient’s privacy and cannot compromise patient anonymity. (DOCX 19 kb) [file 12879_2016_2144_MOESM1_ESM.docx]

Supplemental Table 1a

| K.pneumoniae | PIPC/TAZ | CTM | CAZ | CPZ/SBT | CPR | AZT | MINO | IPM/CA | MEPM | CPFX | LVFX | AMK | Mark |
| --- | --- | --- | --- | --- | --- | --- | --- | --- | --- | --- | --- | --- | --- |
| K1 | 4 | >64 | 8 | 32 | 64 | 16 | 32 | 2 | <0.5 | 1 | 1 | 2 |  |
| K2 | 2 | 16 | <0.5 | 4 | 4 | 2 | 2 | <0.5 | <0.5 | 2 | 4 | 8 |  |
| K3 | 2 | >64 | 2 | 4 | 16 | 2 | 32 | 1 | <0.5 | <0.5 | 1 | 1 |  |
| K4 | 2 | >64 | 2 | 4 | 16 | 2 | 32 | 1 | <0.5 | <0.5 | 1 | 1 |  |
| K5_1 | 4 | >64 | 4 | 4 | 32 | 4 | 32 | 1 | <0.5 | <0.5 | 1 | 2 |  |
| K5_2 | 8 | >64 | 2 | 8 | 32 | 4 | >32 | 1 | <0.5 | <0.5 | 1 | 1 |  |
| K6_1 | 64 | >64 | 64 | 32 | 16 | 64 | >32 | 1 | <0.5 | >64 | >64 | 1 |  |
| K6_2 | >64 | >64 | >64 | 16 | 16 | 32 | >32 | <0.5 | <0.5 | >64 | 64 | 1 |  |
| K6_3 | >64 | >64 | >64 | 16 | 32 | >64 | >32 | <0.5 | <0.5 | 64 | 64 | 1 |  |
| K7 | 4 | >64 | 4 | 8 | 32 | 4 | 32 | <0.5 | <0.5 | <0.5 | 1 | 1 |  |
| K8 | 2 | >64 | 2 | 2 | 16 | 4 | 16 | 1 | <0.5 | <0.5 | 1 | 1 |  |
| K9_1 | >64 | >64 | >64 | 16 | 16 | 16 | 32 | <0.5 | <0.5 | 2 | 1 | 1 |  |
| K9_2 | >64 | >64 | >64 | 16 | 16 | 16 | 32 | <0.5 | <0.5 | 2 | 1 | 1 |  |
| K10 | 1 | 32 | <0.5 | 4 | 8 | 2 | 2 | <0.5 | <0.5 | 4 | 4 | 8 | © |
| K11 | 2 | 64 | 1 | 4 | 8 | 1 | >32 | <0.5 | <0.5 | <0.5 | 1 | 1 | © |

Supplemental Table 1b

| E. coli | PIPC/TAZ | CTM | CAZ | CPZ/SBT | CPR | AZT | MINO | IPM/CA | MEPM | CPFX | LVFX | AMK | Mark |
| --- | --- | --- | --- | --- | --- | --- | --- | --- | --- | --- | --- | --- | --- |
| E1_1 | 4 | >64 | 4 | 32 | 64 | 16 | 4 | <0.5 | <0.5 | 64 | 32 | 2 |  |
| E1_2 | 4 | 64 | 4 | 32 | 64 | 16 | 8 | <0.5 | <0.5 | 64 | 32 | 2 |  |
| E2 | 2 | >64 | 1 | 4 | >64 | 4 | 4 | <0.5 | <0.5 | 8 | 4 | 2 |  |
| E3 | 1 | >64 | 1 | 8 | >64 | 8 | 8 | <0.5 | <0.5 | 32 | 16 | 2 |  |
| E4_1 | 16 | >64 | 4 | 32 | >64 | 16 | >32 | <0.5 | <0.5 | 64 | 32 | 1 |  |
| E4_2 | 2 | >64 | 1 | 8 | 16 | 4 | 16 | <0.5 | <0.5 | 8 | 8 | 2 |  |
| E4_3 | 2 | >64 | 1 | 8 | 16 | 4 | 4 | <0.5 | <0.5 | <0.5 | <0.5 | 2 |  |
| E4_4 | 2 | >64 | 1 | 8 | 16 | 4 | 32 | <0.5 | <0.5 | 4 | 4 | 4 |  |
| E5 | 2 | 32 | 4 | 2 | 16 | 16 | 2 | <0.5 | <0.5 | 32 | 16 | 2 |  |
| E6 | 1 | 64 | 4 | 2 | 32 | 8 | 2 | 1 | <0.5 | 16 | 8 | 2 |  |
| E8_1 | 16 | >64 | 2 | >32 | 64 | 16 | 32 | <0.5 | <0.5 | 64 | 32 | 2 | © |
| E8_2 | 16 | >64 | 2 | >32 | 64 | 16 | 32 | <0.5 | <0.5 | 64 | 32 | 2 | © |
| E8_3 | 16 | >64 | 2 | >32 | 64 | 16 | 32 | <0.5 | <0.5 | 64 | 32 | 2 | © |
| E8_4 | >64 | >64 | 4 | >32 | >64 | 16 | >32 | <0.5 | <0.5 | 64 | 32 | 2 | © |
| E9 | 16 | >64 | 64 | 32 | >64 | 64 | >32 | <0.5 | <0.5 | >64 | 64 | 2 | © |
